# Comparative Short-Term Clinical Outcomes of Mediastinum Tumor Excision Performed by Conventional VATS and Single-Port VATS: Is It Worthwhile?

**DOI:** 10.1097/MD.0000000000001975

**Published:** 2015-11-13

**Authors:** Ching-Feng Wu, Diego Gonzalez-Rivas, Chih-Tsung Wen, Yun-Hen Liu, Yi-Cheng Wu, Yin-Kai Chao, Ming-Ju Hsieh, Ching-Yang Wu, Wei-Hsun Chen

**Affiliations:** From the Division of Thoracic and Cardiovascular Surgery, Department of Surgery, Chang Gung University Memorial Hospital, Taoyuan, Taiwan (CFW, CTW, YHL, YCW, YKC, MJH, CYW, WHC); and Minimally Invasive Thoracic Surgery Unit (UCTMI); Department of Thoracic Surgery, Coruña University Hospital, Coruña, Spain (GRD).

## Abstract

Single-port video-assisted thoracoscopic surgery (VATS) has been widely applied recently. However, there are still only few reports describing its use in mediastinum tumor resection. We present the technique of single-port video-assisted thoracoscopic mediastinum tumor resection and compare it with conventional VATS with regard to short-term outcome.

We retrospectively enrolled 105 patients who received mediastinum surgery in Chang Gung Memorial Hospital. Sixteen patients received sternotomy or thoracotomy, 29 patients received single-port VATS, and 60 patients received conventional VATS (3 ports). The operative time, blood loss, postoperation day 1 pain score, discharge day pain score, and postoperative hospital stay were compared. In order to establish a well balanced cohort study, we also use propensity scores match (1:1) to compare the short-term clinical outcome in 2 groups.

No operative deaths occurred in this study. Single-port VATS was associated with shorter operative time, lower postoperation day 1 pain score, and shorter postoperation hospital stay in our cohort study (*P* = 0.001, <0.001, and 0.039), and propensity scores matched cohort study (*P* = 0.003, <0.001, and <0.001).

Single-port VATS for mediastinum tumor appears to be a safe and promising technique with short-term outcome not inferior to conventional VATS in our cohort study. The long-term oncology outcome may require time and more enrolled patients to be further evaluated.

## INTRODUCTION

In surgery for mediastinal lesion resection, video-assisted thoracoscopic surgery (VATS) is widely used for the resection of a mediastinal mass without invasion to major vessels or organs.^1–4^ Since 2004, single-incision thoracoscopic surgery has been reported, but for a time its use was limited to wedge resection^5,6^ until 2010, when Gonzalez-Rivas et al^7^ described their first experiences of single-port thoracoscopic lobectomy and tried to expand its use in chest surgery. However, to date few studies have mentioned single-port VATS mediastinum tumor resection.^8^ Therefore, we reviewed our series of cases with a mediastinum tumor removed by VATS and compared the short-term clinical outcome of single-port VATS with conventional VATS.

## PATIENTS AND METHODS

**FIGURE 1:**
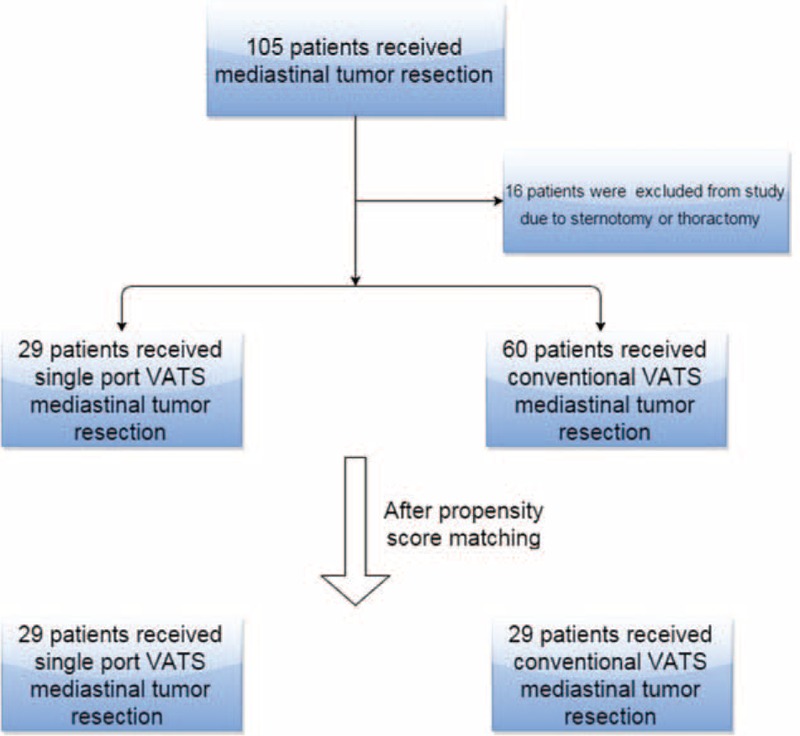
Flow diagram of patient recruitment.

From January, 2013 to April, 2015, 105 patients with a mediastinal tumor underwent operation at the Department of General Thoracic Surgery at Chang Gung Memorial Hospital. Sixteen patients with tumor invasion to major vessels and who received thoracotomy or sternotomy operations were excluded from this study. Among the other 89 patients, 29 patients received single-port VATS operations and 60 patients received conventional VATS operations (3 ports VATS) (Fig. 1). Written informed consent was obtained from all patients before the operation. The preoperative workup included chest radiography, chest computed tomography, spirometry, complete blood counts, and so on. Age, sex, results of pulmonary function tests, Myasthenia Gravis (MG) history, operation time, blood loss, postoperative complications, length of hospital stay, and tumor characteristics were collected from the hospital information system. Surgical mortality was defined as death occurring during the same hospitalization or within 30 days after the operation. In our study, the criteria for single-port VATS and conventional VATS were the same. The indications for thoracoscopic surgery included mediastinal tumor but where no neoadjuvant therapy was given; mediastinal tumor had not directly invaded surrounding tissue, as seen under computed tomography scan; and symptomatic mediastinum cyst or patients were referred to surgical intervention for tissue proof and tumor excision. Whether patients received single-port VATS or not depended on the individual operators. In our department, some operators always adopt single-port technique for their patients and others choose conventional VATS for their patients.

### Surgical Technique

**FIGURE 2:**
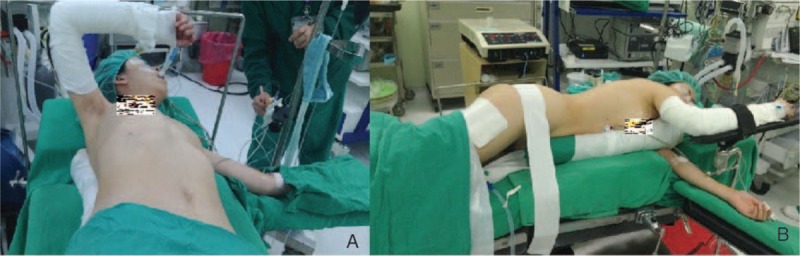
(A) Semisupine position for anterior mediastinum tumor (B) semiprone position for posterior mediastinum tumor.

**FIGURE 3:**
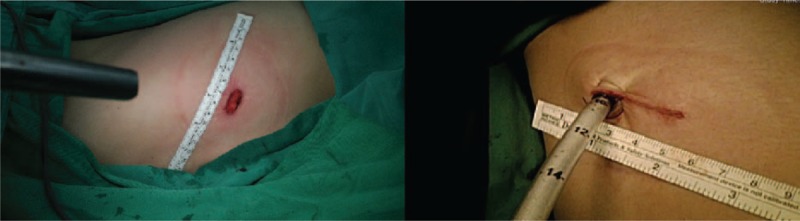
Drainage tube was left for drainage.

The operative technique of single-port VATS was as follows: if patients had anterior mediastinum tumor, the patients were positioned in a 30° semisupine position on the operating table with a roll placed beneath the shoulder and the ipsilateral arm held abducted over a padded L-screen to expose the axilla (Fig. 2A). If the patients had posterior mediastinum tumor, they were placed in the semiprone position, with the contralateral hand placed beneath the neck and the ipsilateral side of the chest elevated by approximately 30°. The ipsilateral arm was raised cranially to expose the axillary fossa (Fig. 2B). If the tumor was located at the middle mediastinum, the traditional VATS position was used. Lung isolation was obtained with a double lumen endotracheal tube ventilation. A 2- to 3-cm wound was created in the 4th or 5th intercostal space at the anterior axillary line. A 30° 5 or 10 mm thoracoscope was then placed at the top of the incision wound. Rib resection or rib spreading was not used in our experience. All procedures were performed under thoracoscope. The mediastinum specimen was retrieved by a plastic bag through the incision wound. Three ports VATS was applied in 60 cases, whereby the patients’ preparation was the same as for single-port VATS. The differences between single VATS and 3 port VATS are that: we create 3 wounds, 1 for a 10-mm 30° thoracoscope and 2 working ports for the endoscopic instruments. The specimen is retrieved by a plastic bag from the anterior working port. At the end of the surgery, 1 chest tube or pig-tail is left for drainage. The drainage tube is placed at 1 end of the incision wound (Fig. 3). Whether a chest tube or pig-tail is left for drainage depends on the operator's decision.

### Visual Analog Scale Score

**FIGURE 4:**
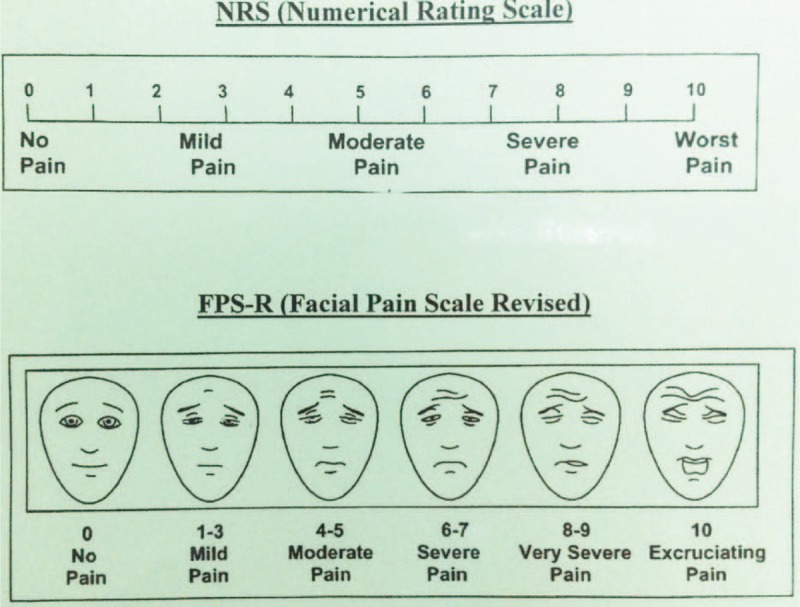
Visual analog scale (VAS) score chart card.

The intensity of postoperative pain was determined using a visual analog scale score.^9^ We used a chart card with a 10-cm horizontal line with word anchors at each end, ranging from 0 = “no pain” to 10 = “worst pain.” If the patients had difficulty communicating with us directly, we used the same chart card with pain scaled facial pictures to evaluate the pain severity (Fig. 4). The pain score was recorded in our hospital information system in the daily progress note.

### Statistical Analysis

**FIGURE 5:**
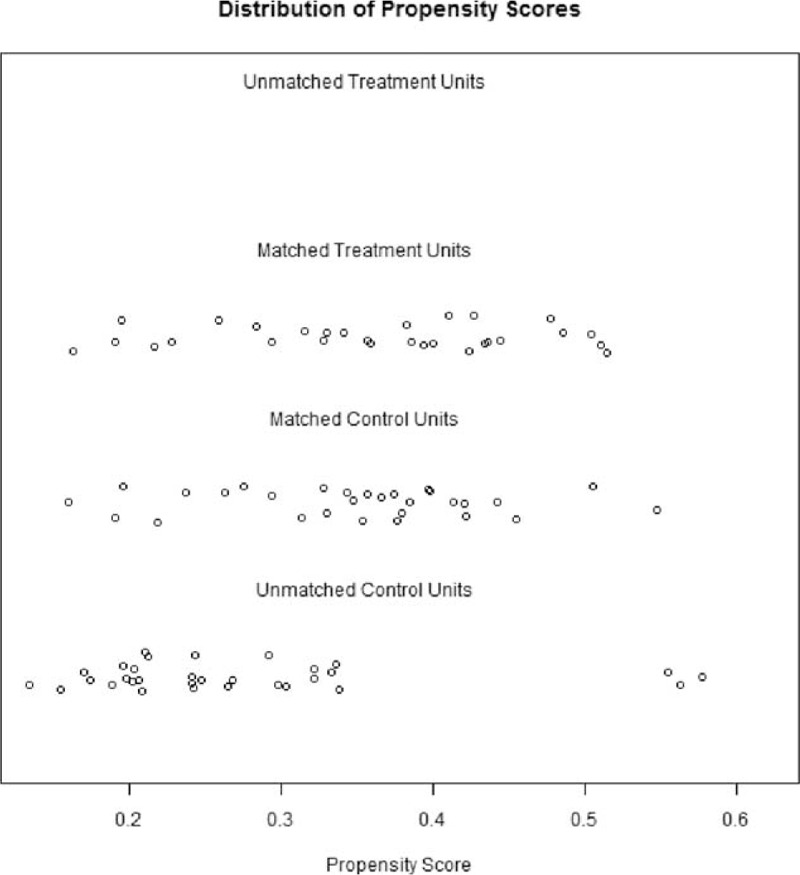
Distribution of propensity scores match.

Continuous data are expressed as mean value with a range of 1 standard deviation (SD). We compared the single-port VATS group and conventional VATS group's operation time, operative blood loss, postoperation day 1 pain score, discharge day pain score, and postoperation hospital stay by one way ANOVA. Before comparison, the Levene test was used to assess the equality of variances. If the variances were not equal, we used the Brown–Forsythe test to see whether any significant difference existed between the 2 groups. For a more accurate comparison of the difference between the 2 groups, we also used a propensity score match (1:1) for the 2 groups.^10^ The propensity score model was generated using all potential covariates that could affect the group allocation; aiming to obtain more reliable results. We included baseline characteristics (age, gender, body mass index, forced expiratory volume in one second, tumor diameter, mediastinum tumor location: anterior, middle, or posterior, and tumor characteristics: cyst or solid mass) for propensity score matching (Fig. 5). After propensity score matching, the general characteristics of the study groups were compared using the one way ANOVA or Brown–Forsythe test, as appropriate. Two-tailed *P* values of 0.05 were considered statistically significant. All calculations were performed using the SPSS statistical package, version 19.0 (SPSS Inc., Chicago, IL) and R 2.12

## RESULT

The demographic data of the 2 groups are presented in Table 1. A total of 53 female and 36 male patients were enrolled (median age 52 years; range 20–85 years). The mean tumor sizes were 4.08 and 4.02 cm in the single-port and conventional VATS groups, respectively. There was no 30 day mortality in either group. We analyzed the 2 groups’ operation time, operation blood loss, postoperation day 1 pain score, and postoperation hospital stay, and we found there was no difference in operative blood loss (*P* = 0.554) and discharge pain score (*P* = 0.110), but the mean operation time, postoperation day 1 pain score, and postoperation hospital stay were lower in patients undergoing single-port VATS than in those undergoing conventional VATS, respectively (*P* = 0.001, <0.001, and 0.039).

**TABLE 1:**
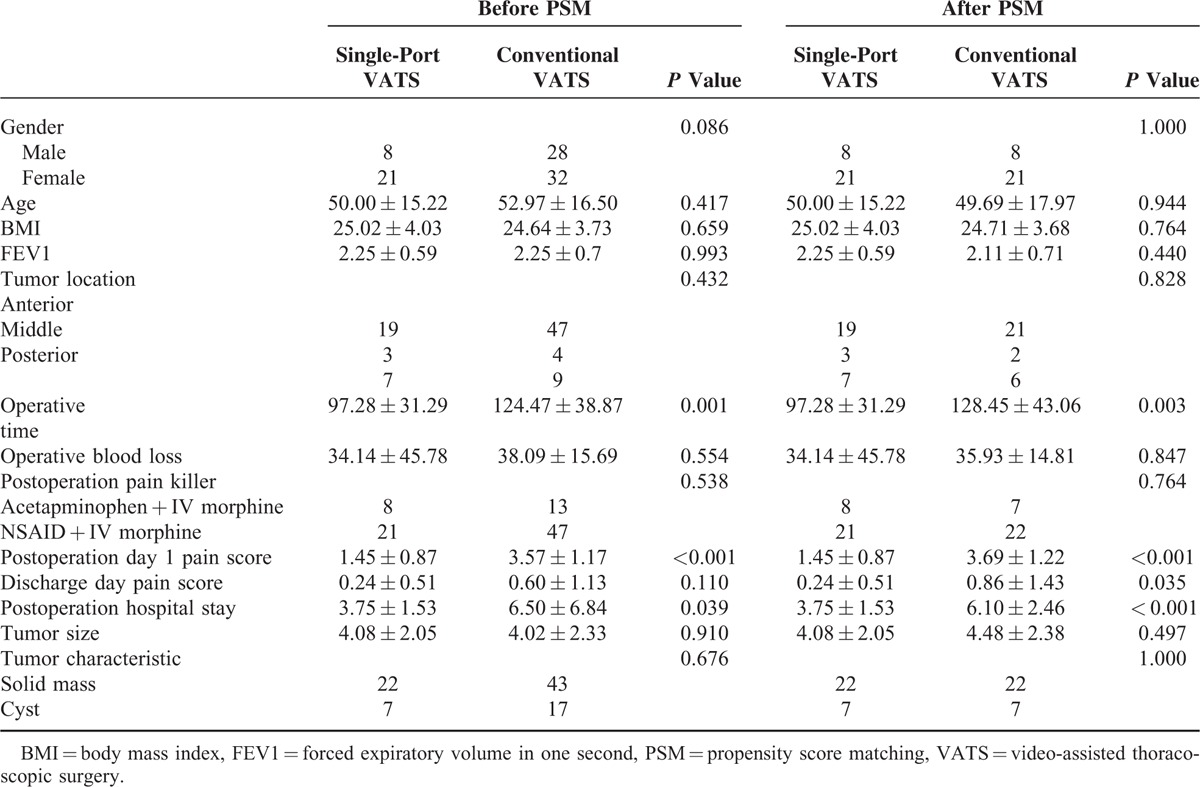
Demographic and Perioperative Features of the Study Patients

**FIGURE 6:**
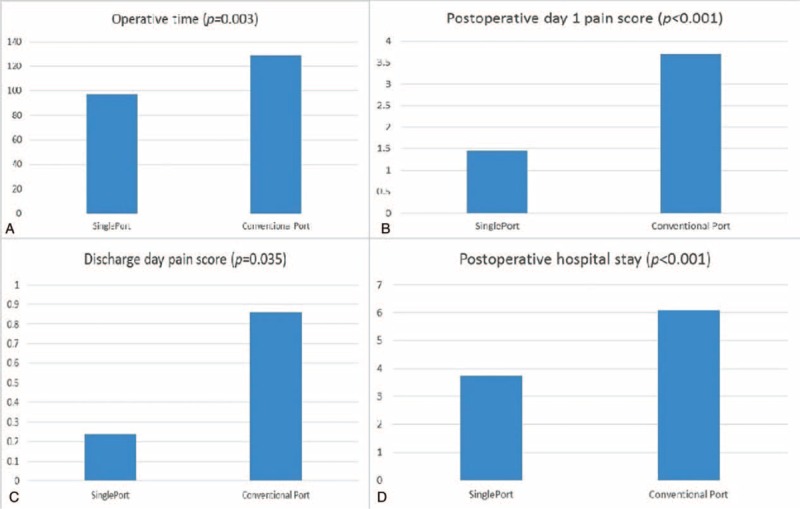
Comparisons between single-port and multiport group: (A) operative time, (B) postoperation day 1 pain score, (C) discharge day pain score, and (D) postoperation hospital stay after propensity scores matching.

For a more accurate comparison of the 2 groups, we used a propensity score based on 7 variables (gender, age, body mass index, forced expiratory volume in one second, tumor diameter, mediastinum tumor location, and tumor characteristics: cyst or solid tumor). Each patient in the single-port VATS group was matched to a patient in the conventional VATS group having the same propensity score, ultimately resulting in a 1-to-1 matched sample cohort. We found postoperation hospital stay was significantly shorter in the single-port VATS group (3.75 days) than in the conventional VATS group (6.10 days; *P* < 0.001). Operative time was shorter in the single-port VATS group (97 vs 128 minutes *P* = 0.003), and postoperation day 1 pain score and discharge pain score were also lower in the single-port VATS group (1.45 vs 3.69, *P* < 0.001 and 0.24 vs 0.86, *P* *=* 0.035 Fig. 6).

Prolonged intubation occurred in 5 patients (2 in the single-port VATS groups and 3 in the conventional VATS group). Prolonged intubation was defined as an intubation time longer than 48 hours. These all 5 patients had a history of MG.

## DISCUSSION

After its introduction in the 1990s, VATS was expected to become the procedure of choice not only for benign mediastinal tumors^3,11^ but also for noninvasive thymoma.^1,2,4^ Proponents of minimally invasive surgery have emphasized its benefits, including less blood loss in the operation, less pain in the early postoperative period, less compromised pulmonary function, and better cosmetic results.^12–14^ Single-port VATS is a new technique that has developed rapidly recently.^7,15^ Safety is the most important consideration when a new surgical method is developed, and it is important to note that no intraoperative or immediate postoperative complications occurred in either group in our study. However, longer follow-up is needed to evaluate postoperative complications fully and a larger sample size would be needed to provide robust data.

Whether single-port VATS is superior to conventional VATS in postoperative pain management remains an open issue^16–18^ and most articles discuss lung resection and pneumothorax. This paper focused on mediastinal tumor resection only. In order to get a more clear result, we used propensity score matching to minimize the difference between the 2 groups to see whether single-port VATS is superior to conventional VATS or at least not inferior. In our well-balanced cohort, we did indeed find that patients had less pain sensation, which may result in better daily activity and shorter postoperation hospital stay. Surprizingly, we also found no difference in operative blood loss between the 2 groups and operative time was shorter in the single-port VATS group. However, this does not justify the conclusion that single-port VATS is better than conventional VATS; for this, the present study was underpowered. We could only conclude that single-port VATS is a promising technique if operators can conquer its learning curve and instrument “fencing,” where long endoscopic instruments interfere with each other.

With regard to oncological concerns, the occurrence of postoperative MG or tumor recurrence is a paramount issue. Our cohort follow-up time was not long enough to answer this question, and more time and a greater number of patients are needed for further evaluation. Some caveats of the current study merit comment. First, although propensity matching may reduce the bias inherent in a comparison of 2 different surgical techniques, future prospective, randomized trials are needed to confirm our findings. Second, limited case numbers may also result in statistical bias. Thus, further study should be conducted with more patients. Third, the pain score evaluation was subjective and may be affected by patients’ psychological condition. A more complete questionnaire survey may be necessary to assess more accurately patients’ true experience of pain.

## CONCLUSION

In conclusion, in our cohort study of patients without tumor invasion to major organs or stage I and II thymoma, single-port VATS appears to be a safe and promising technique, associated with a shorter hospital stay and less postoperative pain than conventional VATS. The long-term oncological outcomes need further evaluation.
